# Brief Effect of a Small Hydrophobic Drug (Cinnarizine) on the Physicochemical Characterisation of Niosomes Produced by Thin-Film Hydration and Microfluidic Methods

**DOI:** 10.3390/pharmaceutics10040185

**Published:** 2018-10-13

**Authors:** Li Key Yeo, Temidayo O. B. Olusanya, Cheng Shu Chaw, Amal Ali Elkordy

**Affiliations:** School of Pharmacy and Pharmaceutical Sciences, University of Sunderland, Sunderland SR1 3SD, UK; li-key.yeo@research.sunderland.ac.uk (L.K.Y.); temmywood@yahoo.com (T.O.B.O); cheng.chaw@sunderland.ac.uk (C.S.C.)

**Keywords:** niosome, cinnarizine, poorly water-soluble drug, thin-film hydration, microfluidic, co-surfactants

## Abstract

Novel niosomal formulations containing cinnarizine were developed to enhance its drug characteristics. In this work, niosomes (non-ionic surfactant vesicles) were prepared by conventional thin-film hydration (TFH) and microfluidic (MF) methods with sorbitan monostearate (Span^®^ 60), cholesterol, and co-surfactants (Cremophor^®^ ELP, Cremophor^®^ RH40 and Solutol^®^ HS15) as key excipients. The aim was to study the effect of cinnarizine on the characteristics of different niosomal formulations manufactured by using different methods. For effective targeted oral drug delivery, the efficacy of niosomes for therapeutic applications is correlated to their physiochemical properties. Niosome vesicles prepared were characterised using dynamic light scattering technique and the morphology of niosomes dispersion was characterised using optical microscopy. Dialysis was carried out to purify niosome suspensions to determine drug loading and drug release studies was performed to study the potential use of niosomal systems for cinnarizine.

## 1. Introduction

Niosomes are non-ionic surfactant-based bilayer membrane vesicles that are formed by self-assembly upon hydration. Non-ionic surfactants are amphiphilic molecules that are biodegradable, biocompatible, and non-immunogenic. Niosomes are capable of entrapping both hydrophobic and hydrophilic drugs [1]. They have been successfully manufactured for oral delivery of cytotoxic agents such as paclitaxel [2] at a lower cost with various surfactant combinations by film hydration method. The potential use of niosomes as an oral delivery system has been studied to overcome the challenges of limited absorption due to poor drug stability and poor water solubility in the gastrointestinal tract [3,4]. Niosomes are similar to liposomes (which are comprised of phospholipids) as drug delivery systems. Unlike liposomes, niosomes have less chemical instability problems, but they are associated with physical stability issues such as fusion, aggregation, sedimentation, and drug leakage during storage [3]. Numerous studies have shown that niosomes have a high potential as a carrier for poorly water-soluble drugs such as paclitaxel [2], valsartan [5], candesartan [6], lornoxicam [7], diacerein [8], griseofulvin [9], flurbiprofen [10], and diclofenac [11]. 

Spans are the product name marketed for sorbitan esters; they are produced by the dehydration of sorbitol. The hydrophile-lipophile balance (HLB) value of Span decreases with increasing the length of the alkyl chain (increasing the number of fatty acid groups); for example, sorbitan monostearate (Span^®^ 60) has an HLB value of 4.7 and sorbitan tristearate (Span^®^ 65) has an HLB value of 2.1; sorbitan monooleate (Span^®^ 80) has an HLB value of 4.3, and sorbitan trioleate (Span^®^ 85) has an HLB value of 1.8.

Their gel transition temperature increases as the length of the acyl chain increases resulting in decreased leakage of drugs from niosomes [12]. All Spans have the same head group and different alkyl chain length [13]. As the alkyl chain length increases, the entrapment efficiency of hydrophobic drugs is expected to increase. The encapsulation efficiency (%EE) is decreasing from Span^®^ 60 (C_18_), Span^®^ 40 (C_16_), Span^®^ 20 (C_12_), and Span^®^ 80 (unsaturated C_18_). This was in an agreement with flurbiprofen pro-niosomes studied by Mokhtar et al. [10]. In addition, the study found that with increasing total lipid or drug concentration use resulted in an increased %EE of the flurbiprofen produced. Sorbitan monostearate (Span^®^ 60) with a C_18_ chain has a gel transition temperature of 56–58 °C and an HLB value of 4.7, exhibits the highest entrapment efficiency, therefore it was chosen for this current study. The molar ratio of cholesterol incorporated with surfactants may affect the entrapment of drugs into niosomes. Nasseri [14] reported that the equimolar mixture (Span^®^ and cholesterol) represented the critical composition as there is only one hydrogen bonding group on the cholesterol moiety to interact with oxygen functionalities on the Span^®^ 60, resulting in an increase in membrane cohesion.

The incorporation of cholesterol with surfactants of lower HLB values has shown to promote the gel liquid transition temperature of the vesicle [15]. Kumar and Rajeshwarrao [12] reported the addition of cholesterol enables more hydrophobic surfactants (lower HLB values) to form niosomes by suppressing the tendency of aggregation. Cholesterol helps by increasing the orientation order of their relatively mobile hydrocarbon chains of liquid-crystalline phospholipid bilayers, decreasing bilayer permeability and reducing the efflux of the entrapped drug. This resulted in prolonged drug retention [16] and effectively prevented leakage of the drug from niosomes [13].

Other additive agents to enhance the characteristics of the niosome vesicles are co-surfactants. Co-surfactants are usually more hydrophilic; therefore, they act as emulsifying, solubilizing, and wetting agents as they have generally a higher HLB value from 12 to 16 and a higher molecular weight of over 1000 Da. Co-surfactants are commonly used in the literature for niosome formulations, for example Cremophor^®^ ELP (purified polyoxyl 35 castor oil) [17], Cremophor^®^ RH40 (hydrogenated polyoxyl 40 castor oil) [18], and Solutol^®^ HS15 (polyoxyl 15 hydroxystearate) [19]. Solutol^®^ HS15 is known to inhibit P-glycoprotein, which is an ATP-dependent pump that is responsible for reducing drug intestinal absorption by efflux transportation. Addition of this agent has shown to enhance paclitaxel aqueous solubility and permeability across Caco-2 monolayer cell without inducing cytotoxicity [17]. This would further enhance oral bioavailability of drugs such as cinnarizine (in this study) in the niosomal formulations.

There are numerous studies which have investigated the preparation of niosomes using the conventional thin-film hydration method and the reverse phase evaporation technique. The formation of film by evaporation of organic solvent is followed by hydration to produce multi-lamellar vesicles which are non-reproducible and a post size reduction process is needed to generate homogenous vesicles [18]. Compared to the conventional bulk method, microfluidic technique has been used to prepare lipid-based nanoparticles and liposomes to generate reproducible small-sized nanoparticles for drug encapsulation. Correia et al. [19] reported that microfluidic systems step up in the area of drug delivery with promising features that allow control of particle size and increased stability of the final liposome products. Hence, this technique will be used in this current research for niosome production.

Cinnarizine is a piperazine derivative with antihistaminic, sedative, antiserotonergic, antidopaminergic, and calcium channel blocking activities [20]. It is also used as cerebral blood flow improver in the management of various peripheral and cerebral vascular disorders. Cinnarizine is a lipophilic weak base (literature pKa values of 1.95 and 7.47) and a log *P* of 5.6 [21]. The drug is practically insoluble in water in its unionized form and has a narrow absorption window. After oral administration of conventional tablet formulations, absorption is relatively slow, peak serum concentrations occurring after 2.5 to 4 hours [22] from the upper gastrointestinal tract. This is due to patients’ gastric acidity influence on the dissolution and absorption of cinnarizine.

The study was aimed to develop and characterize cinnarizine-containing niosomes prepared by thin-film hydration and microfluidic methods, with the future aim to coat the niosome vesicles manufactured with a muco-adhesive agent (chitosan) for a prolonged drug retention in the stomach (to enhance drug bioavailability). To the best of our knowledge, there are no literature available for formulation of cinnarizine in niosome systems using either thin-film hydration or microfluidic methods, hence it is worth to characterize niosomes with this hydrophobic drug.

## 2. Experimental Section

### 2.1. Materials

Cinnarizine, Span^®^ 60 (S60), cholesterol (Cho), phosphate buffered saline (PBS) tablets, chloroform, ethanol, hydrochloric acid 37%, and isopropanol were purchased from Sigma-Aldrich (Dorset, UK). Cremophor^®^ ELP (ELP), Cremophor^®^ RH40 (RH40), and Solutol^®^ HS15 (HS15) were obtained from BASF (Cheshire, UK). All materials and chemicals were of analytical grade and used as received.

### 2.2. Preparation of Niosomes

In conventional thin-film hydration method, weighed lipids (refer to Table 1) and cinnarizine were dissolved in chloroform and then transferred into a 100 mL round-bottomed flask. The formation of film was produced by using the rotary evaporator (Buchi rotavapor R-210, Flawil, Switzerland) to remove chloroform under pressure of 325 ± 10 mbar at 60 °C in a water bath and 100 revolutions per minute (rpm). Afterwards, the thin film obtained was allowed to dry completely and cooled at room temperature before underwent hydration process with 20 mL of phosphate buffered saline (PBS 10 mM, pH 7.4) solution using water bath shaker at 60 °C and 100 rpm for 30 min to produce niosome suspensions. Size reduction step (e.g., sonication) was excluded to investigate the influence of preparation methods on niosome characteristics.

As for microfluidic method, NanoAssemblr^TM^ Benchtop system (Precision NanoSystems Inc., Vancouver, VAN, Canada) with a microfluidic cartridge and a heating block controller was used. Weighed lipids (refer to Table 1) and cinnarizine (without cinnarizine for empty pre-formed niosomes) were dissolved in ethanol to be used as organic phase and aqueous phase used was PBS. Process parameters applied were flow rate ratio of 4:1 (aqueous:organic), a total flow rate of 12 mL/min, and at 60 ± 1 °C (these conditions have been used based on preliminary studies).

A modified dialysis method from reference [23] was used for all niosome suspensions prepared to be dialysed (using a dialysis membrane, MWCO: 3500 Da) against 1 L of 0.1 M hydrochloric acid solution (HCl, pH 1.2) under magnetic stirring for overnight to remove non-entrapped free drug and any trace of organic solvent.

### 2.3. Morphological Analysis of Niosomes Using Optical Microscope

Freshly prepared niosome suspensions were observed under light microscope with magnification lens of 40× using a MicroCam Olympus BH-2/LB with AxioCam MRc (Carl ZEISS, Jena, Germany). The formations of niosomes were confirmed by observation under optical light microscope and real-time images were taken. The niosomes formed were all in a distinctive round, circular shape.

### 2.4. Fourier-Transform Infra-Red (FTIR) Spectroscopy

Purified niosome pellets obtained from centrifugation (15,000 rpm for 20 min at 4 °C) were re-dispersed with 5 mL deionised water (TFH-based niosomes) and 5% mannitol (MF-based niosomes), and then kept in an ultra-low freezer (−80 °C) for 2 h prior to the freeze-drying process using Christ Advance Alpha 2–4 LSCplus freeze dryer, Osterode, Germany. The vacuum was set to 0.035 mbar with ice condenser set at −81 °C and shelf temperature at 10 °C. The infrared spectra of individual material and freeze-dried niosome samples were taken using a Shimadzu IRAffinity-1S spectrophotometer (Shimadzu UK Ltd., Buckinghamshire, UK). The spectra were recorded using 12 scans in the wavelength range (4000–550 cm^−1^) with a resolution of 4 cm^−1^ to study their possible interactions using the Shimadzu LabSolutions IR. Under the same conditions, the infrared spectrum of the cinnarizine pure drug was also taken for identification of its principle functional groups.

### 2.5. Thermal Characteristics of Niosomes

A standard mode DSC conditioning was performed at 75 °C for 120 min hold time without a refrigerated cooling system (RCS) (DSC Q1000 TA Instruments, Ghent, Belgium) with empty cell chamber. Afterwards, temperature calibration was carried out using pure indium with the RCS. All freeze-dried niosome samples (refer to Section 2.4) weighed between 2 and 8 mg using Mettler MT5 balance (Mettler Toledo, Leicester, UK) were placed within standard aluminium hermetic pans and lids for DSC runs. DSC runs were performed at a heating rate of 10 °C/min from 25–300 °C. The nitrogen gas flow rate was 50 mL/min with the RCS on. Thermal Universal Analysis 2000 was used to perform analysis of the obtained DSC thermograms. 

### 2.6. Particle Size, Polydispersity, and Charge of Niosomes

An aliquot from each of the freshly prepared niosome suspensions was used for measurement of particle size, polydispersity index (PDI), and zeta potential (ZP) in 1/20 dilution with deionised water, using Malvern Zetasizer Nano ZSP (Cambridge, UK). Measurement angle of 173° backscatter was used for angle of measurement detection. Three measurements were taken for each sample. Hydrodynamic size and PDI were measured using dynamic light scattering (DLS), and the zeta potential was determined using laser Doppler electrophoresis. The PDI is an indicator for particle size distribution ranging from 0 (narrow distribution) to 1 (wide distribution). 

### 2.7. Measurement

#### 2.7.1. Calibration Curve

Cinnarizine standard solutions (10 to 500 ng/mL pure cinnarizine in 0.1 M HCl solution, pH 1.2) were prepared for calibration measurement using reverse phase ultra-high performance liquid chromatography (uHPLC). The HPLC system (Agilent technologies, Waldbronn, Germany) consisted of Agilent Chem Station LC-DAD with UV detector. The method was developed using Agilent Zorbax Eclipse Plus C18 (50 mm × 2.1 mm × 1.8 µm) column with gradient system of mobile phases consisting of 0.1% formic acid in water as mobile phase A and 0.1% formic acid in acetonitrile as mobile phase B (see Table 2). The flow rate was maintained at 0.3 mL/min. The column temperature was maintained at 40 °C and the detection wavelength used at 255 nm. The injection volume used was 10 µL for all measurements (standard solutions and samples). Agilent ChemStation software was used to record and integrate responses of peak areas and retention time.

#### 2.7.2. Drug Encapsulation Efficiency and Drug Release Study

**Figure 1:**
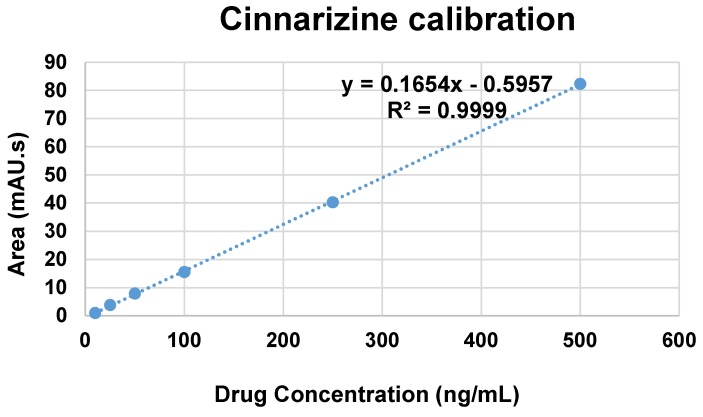
Cinnarizine calibration curve.

Purified niosome suspensions and their respective dialysates were assayed for the encapsulation efficiency, %EE. Isopropanol was used to disrupt the purified niosomes and filtered using 0.45 µm syringe filter prior to HPLC measurements for quantification. %EE was calculated as the percentage of total drug content (entrapped cinnarizine) after excluding free drug (non-entrapped cinnarizine) and based on the calibration equation obtained (Figure 1).

A modified method [24,25] for in vitro drug release study was performed using dialysis membrane containing 5 mL of the purified niosome suspension (MF-based S60:Cho:ELP) immersed in a beaker containing 200 mL of 0.1 M HCl solution (pH 1.2) as dissolution medium. The beaker was placed in a water bath shaker at 37 °C and 50 rpm. At predetermined intervals, 1 mL of the dissolution medium was withdrawn and then replaced with 1 mL of fresh 0.1 M HCl solution. The drug contents in the dissolution medium were calculated using the HPLC measurements based on the calibration equation obtained.

## 3. Results and Discussion

### 3.1. Morphological Analysis of Niosome Suspensions

**Figure 2:**
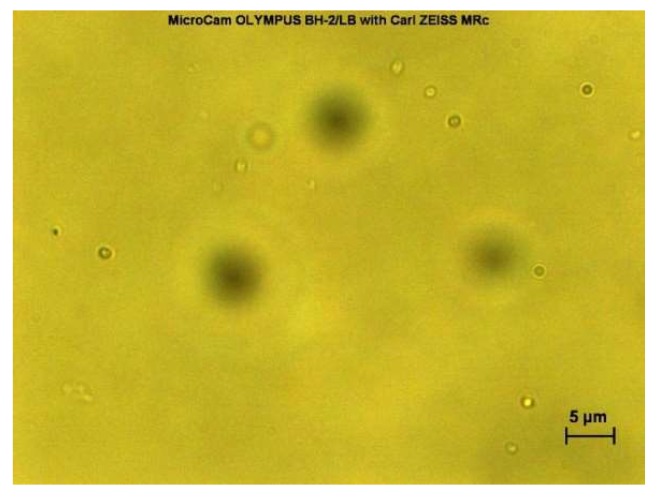
Microfluidic-prepared empty niosomes comprised of Span^®^ 60, cholesterol, and Cremophor^®^ ELP.

**Figure 3:**
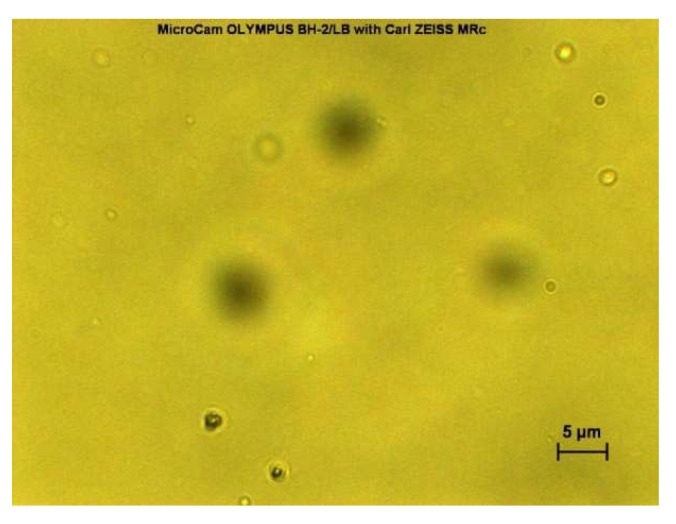
Microfluidic-prepared cinnarizine-containing niosomes comprised of Span^®^ 60, cholesterol, and Cremophor^®^ ELP.

The formation of freshly prepared niosomes for both film hydration (result not shown) and microfluidic (see Figure 2 and Figure 3) methods were confirmed by observation under light microscope with magnification lens of 40× using Olympus BO61 (AxioCam MRc ZEISS) and real-time images were taken. Both blank niosomes and niosomes containing cinnarizine were formed successfully upon hydration by distinctive round, circular shapes observed.

### 3.2. Particle Size, Polydispersity, and Charge of Niosomes

Hydrodynamic parameters apply to particles in dispersion or molecules in solution. Dynamic light scattering (DLS) is a low-resolution technique, requiring a 3 fold difference in size in order to achieve baseline resolution in the particle size distribution [26]. By using thin-film hydration method (Table 3), the niosome dispersed in the formulations without the inclusion of non-ionic co-surfactants, which did not form successfully or produce large aggregates as compared to formulations with a co-surfactant incorporated. Although there are studies that indicate the formation of niosomes using Span^®^ 60 and cholesterol in 1:1 molar ratio without co-surfactants, for example Yoshioka et al. [25]; however, the explanation of our results is that the other studies in addition to applying different preparation conditions (e.g., hydration time and/or shaking speed) they used different model drugs than cinnarizine which was utilised for this current study. 

With cinnarizine dissolved in chloroform during niosome preparation, particle size of both blank and cinnarizine-containing niosomes prepared by TFH method are widely distributed with noticeably higher particle size ranging from 827 to 7320 nm and a higher polydispersity index (PDI) of 0.3 to 0.9 (see Table 3). All cinnarizine-containing niosomes revealed larger particle size compared to blank niosomes (without cinnarizine inclusion). This was in an agreement with the study reported by Taymouri and Varshosaz [27]. In contrast, with cinnarizine dissolved in 0.1 M HCl solution during niosome preparation in the previous preliminary study, cinnarizine-containing niosomes demonstrated a decrease in particle size with drug incorporation compared to empty niosomes. Similarly, particle size distributed widely, ranging from 1 to 9 µm and a wide PDI ranging from 0.1 to 1.0. Generally, multilamellar niosomes are produced by thin-film hydration method and this contributes to a higher particle size distribution without size reduction process.

Non-ionic surfactants are amphiphilic molecules with different surface activity properties that depend on the balance between their hydrophilic and hydrophobic regions. Surfactants with a hydrophile-lipophile balance (HLB) value between 3 and 8 are suitable to form a bilayer-membrane vesicle and are commonly referred to as water-in-oil (W/O) emulsifiers [15]. As non-ionic surfactants have no charge groups in their hydrophilic head region, they are less toxic, more resistant against pH changes in the gut, and with a wider compatibility compared to ionic surfactants. Other than an HLB value of a non-ionic surfactant, the structure of a surfactant has a great impact on the geometry formation of its vesicle attributed to critical packing parameters (CPPs) including the hydrophobic group volume, critical hydrophobic group length, and the area of hydrophilic head group [28]. Larger vesicles are formed when the hydrophilic portion of the molecule is decreased relative to the hydrophobic portion as increasing in the alkyl chain length would result in an increase in the CPP value [29]. As cinnarizine is a hydrophobic drug, it has the tendency to interact and to be entrapped within the hydrophobic regions of the bilayer membrane of the multilamellar vesicles formed by TFH method.

On the other hand, the results obtained in this study revealed that there was interaction between the niosome vesicles and the hydrophobic drug which contributed to the smaller vesicle size. As the drug surface activity is minor compared to the surfactants used, the decrease in vesicles’ size may be due to the presence of the drug which implied a co-surfactant drug effect as described by Nikolakakis et al. [30]. Therefore, cinnarizine incorporation within the niosome vesicles of equimolar of Span^®^ 60 and cholesterol with co-surfactants is shown to decrease the size of niosomes. Cremophor^®^ RH40 as a hydrophilic co-surfactant exhibited the smallest vesicle size, this might be due to larger molecular weight and higher surface activity, as compared to formulations comprised of co-surfactants: Cremophor^®^ ELP or Solutol^®^ HS15. Accordingly, each co-surfactant performs differently based on its structure, HLB, and properties. However, all vesicle size range is generally suitable for oral administration dosage forms.

To the best of our knowledge, there has been little literature investigating the influence of co-surfactant on niosomal formulations without post-preparation size reduction step. Therefore, a size reduction process such as homogenization and sonication has to be carried out post preparation of thin-film hydrated niosome vesicles to ensure homogeneity of niosome dispersions, this additional step has drawbacks such as time consuming, low-cost effectiveness, requiring more characterisation processes and affecting the drug loading.

Regarding the zeta potential of niosomes, a slight difference was found between formulations with and without cinnarizine among the different formulations measured (Table 3 and Table 4). The slight positive charge obtained with cinnarizine entrapped niosomes suggested the basic characteristic of the dissolved cinnarizine due to the presence of amine groups’ ionisation in the aqueous solution.

Because thin-film hydration method produced niosomes with variability with the presence of the drug, it was decided to use a microfluidic system (which is mostly applied for preparation of liposomes) to investigate its feasibility in production of niosomes with consistent properties, taking into account there are very sparse literature for microfluidic technique and niosomes. It was found that, generally, the size of niosome vesicles prepared by microfluidic method was very small and indicating monodisperse suspension in comparison to niosome vesicles prepared by thin-film hydration method. The NanoAssemblr^TM^ Benchtop enables rapid formation of much smaller niosomes by mixing two miscible fluids (aqueous and organic solvents) in a single-step process under focused hydrodynamic flow in the architecture of the microfluidic cartridge which prevents suboptimal mixing and heterogeneous nanoprecipitation of ingredients used, which is in agreement with Obeid et al. [31].

For niosomes prepared by microfluidic method (see Table 4), all formulations revealed noticeably small particle size with a low PDI values (narrow distribution) as measured using zetasizer technique, indicating large unilamellar vesicles (LUVs), and were manufactured successfully using microfluidic method. These results demonstrated the potential use of microfluidic technique to prepare small and uniform niosomes, minimising variable encapsulation efficiency, increasing homogeneity and reproducibility of the formulation. The influence of cinnarizine inclusion with increased niosome sizes can be seen in formulations without co-surfactant (F2) and Cremophor^®^ ELP (F4) compared with respective blank formulations (F1 and F3). In contrast, formulations with Cremophor^®^ RH40 and Solutol^®^ HS15 showed a decrease in particle size with cinnarizine inclusion. The slight change in particle size for both with and without cinnarizine inclusion demonstrated an interaction between the niosome vesicle and the hydrophobic drug.

### 3.3. Fourier-Transform Infra-Red (FTIR) Spectroscopy

**Figure 4:**
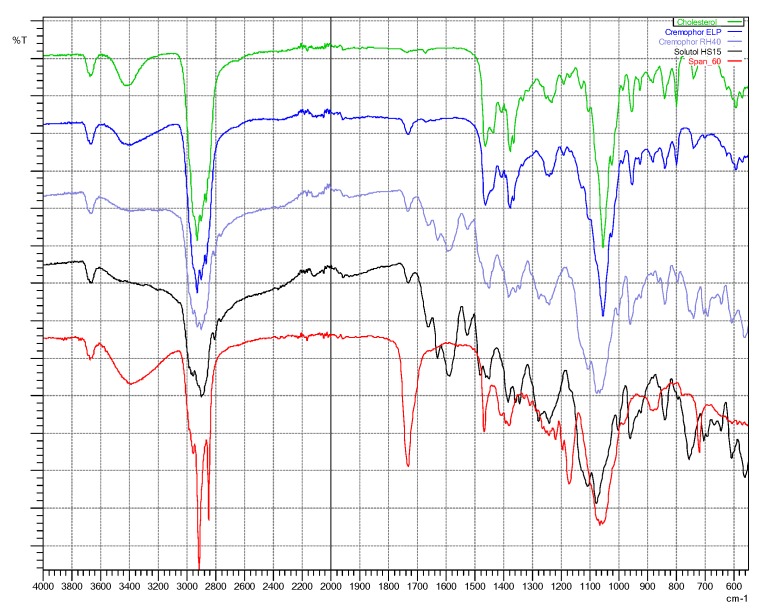
FTIR spectra taken for individual ingredient: cholesterol (green), Cremophor^®^ ELP (blue), Cremophor^®^ RH40 (purple), Solutol^®^ HS15 (black), and Span^®^ 60 (red).

Fourier-transform infra-red (FTIR) spectroscopy is useful for identifying principal peaks of characteristic functional groups of a material as well as determining their changes within a specific finger printing area [32]. In addition, the peak intensity is to establish concentration of a specific bonds or functional groups. Fourier-transform infra-red spectra of individual material (Figure 4) and freeze-dried niosomes were taken to determine their interactions and compatibilities [20]. 

**Figure 5:**
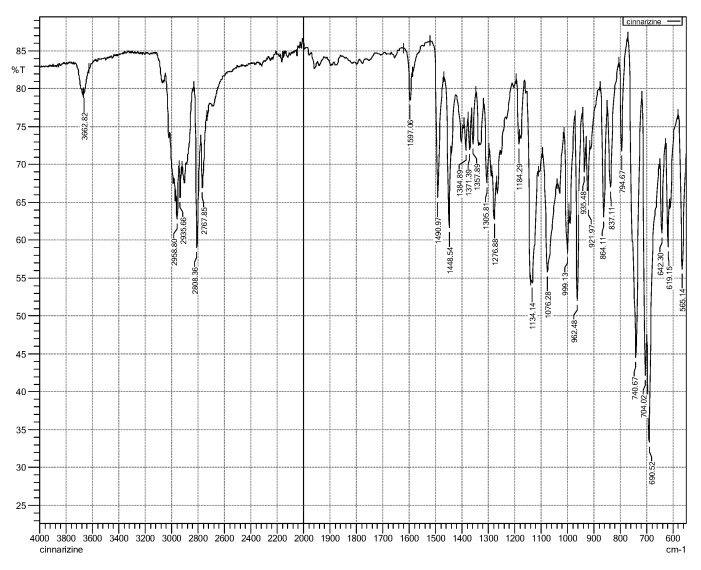
FTIR spectrum of cinnarizine pure drug.

The principle peaks of the cinnarizine pure drug (free base) were observed at 2959, 2936, 1597, 1490, 1448, 1134, 999, and 962 cm^−1^ (see Figure 5). Assignment for the major infra-red absorption bands of cinnarizine (free base pure drug) is shown in the Table 5. 

**Figure 6:**
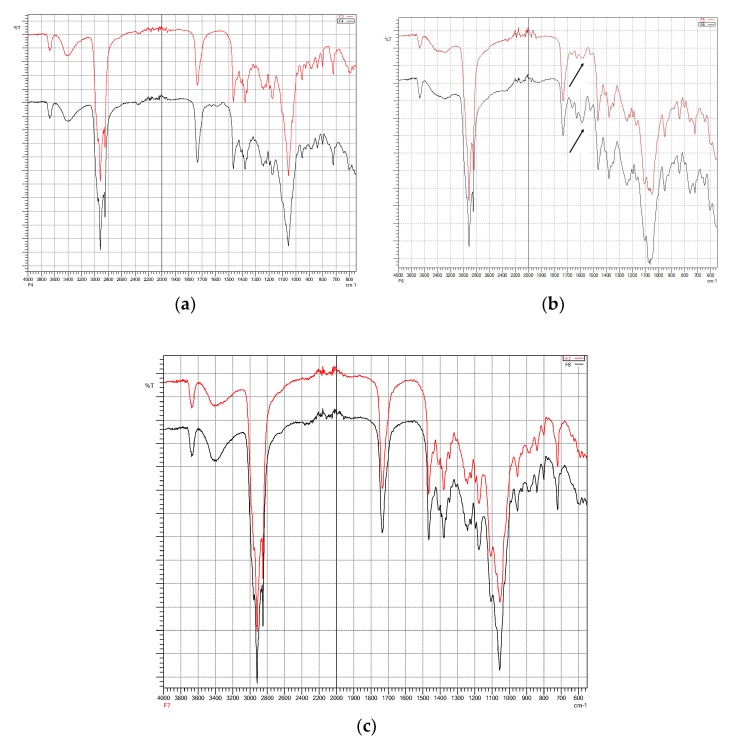
FTIR spectra for (**a**) formulations 3 and 4; (**b**) formulations 5 and 6; (**c**) formulations 7 and 8.

**Figure 7:**
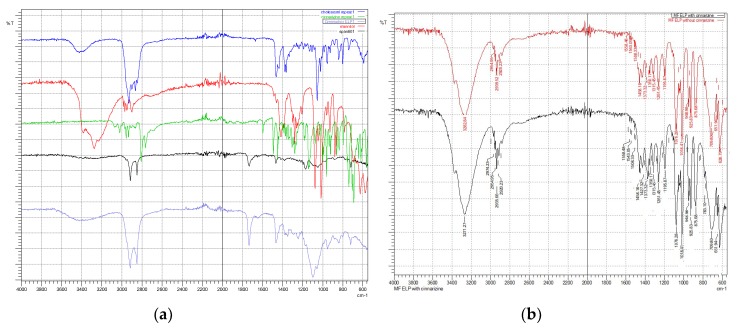
(**a**) FTIR spectra taken for individual ingredient: cholesterol (blue), mannitol (red), cinnarizine (green), Span^®^ 60 (black) and Cremophor^®^ ELP (purple); (**b**) FTIR spectra for MF-based formulations 3 and 4.

Figure 6a–c show a comparison between same formulation composition with and without the presence of cinnarizine. They demonstrate the interaction and incorporation of the drug within the formulations. Similar peaks shown in formulations with and without cinnarizine generally indicate the incorporation of surfactants, cholesterol, and co-surfactants to formation of niosomes. The entrapment of cinnarizine within the vesicles can be seen with the disappearance of C-N stretch (1134 cm^−1^) of the cinnarizine in all formulations (Figure 6). Apart from this C-N stretch, the C=C aromatic stretch of the cinnarizine also disappeared in formulations with Cremophor^®^ ELP (Figure 6a) and Solutol^®^ HS15 (Figure 6c) except for Cremophor^®^ RH40. In Figure 6b, in comparison to Formulation 5 (without cinnarizine), the spectrum of Formulation 6 revealed the presence of cinnarizine with the appearance of its principal peak of C=C stretch, indicating that the drug was not able to be incorporated well within the niosome vesicles. This might be due to the fact that the hydrophilic Cremophor^®^ RH40 has the highest molecular weight among the other solubilising agents, is interacting and inhibiting the incorporation of hydrophobic drug (cinnarizine). Similarly, microfluidic-prepared niosomes (Figure 7a,b) demonstrated the entrapment of cinnarizine into the pre-formed niosomes. 

### 3.4. Thermal Characteristics of Niosomes

**Figure 8:**
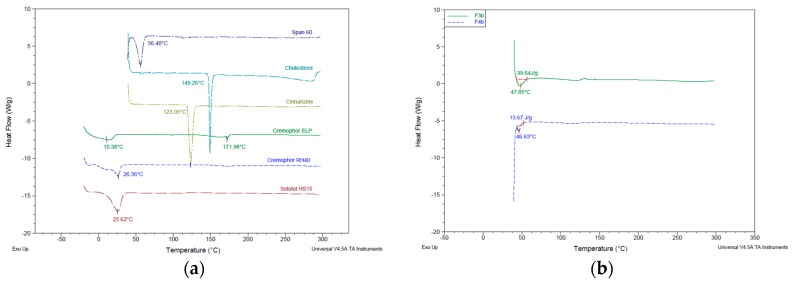

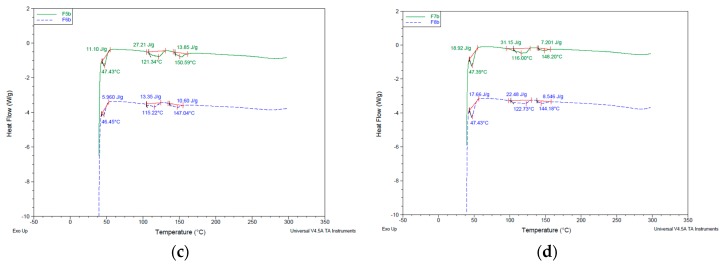
Differential Scanning Calorimetry (DSC) thermographs for (**a**) pure raw materials for niosomes formulation compositions; (**b**) freeze-dried formulations 3 and 4; (**c**) freeze-dried formulations 5 and 6; (**d**) freeze-dried formulations 7 and 8.

**Figure 9:**
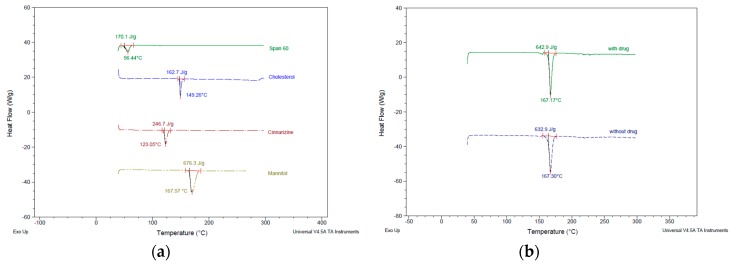
Differential Scanning Calorimetry (DSC) thermographs for (**a**) pure raw materials for niosomes formulation compositions and mannitol as lyoprotectant; (**b**) freeze-dried formulations 3 and 4.

Calorimetry technique in thermal analysis was used to study thermodynamic characteristics (e.g., melting temperature, denaturation temperature, crystallization, and enthalpy change) [33]. In this study, differential scanning calorimetry (DSC) was used to determine the temperatures and heats of transitions for raw materials and freeze-dried niosomes. Heat flow data were obtained as a result of dynamic measurement at a constant heating rate. The DSC curve taken for pure cinnarizine (Figure 8a) showed a sharp endothermic peak at 123.05 °C. This peak either demolished in the freeze-dried niosomes, indicating drug incorporation into niosome vesicles or broaden and shifted to a lower temperature, suggesting changes in the structure arrangement and transformation to amorphous form. Moreover, DSC curves for the freeze-dried thin film hydrated niosomes showed melting endothermic peaks of Span^®^ 60 (shifted from 56 °C to 46–47 °C) and cholesterol (but broaden and shifted from 149 °C to lower temperatures), indicating incomplete compatibility of the excipients used in film hydration method based on the conditions applied. The use of lyoprotectant was reported to preserve the integrity of the vesicles by inhibiting vesicle fusion and aggregation during the freezing stages [4]. The DSC curves in Figure 9 showed the mannitol endothermic peak at 167 °C and the demolition of peaks similar to freeze-dried niosomes without mannitol.

### 3.5. Drug Loading and Drug Release Studies

Low encapsulation efficiencies were found in all formulations prepared by thin film hydration method (<20%; see Table 6) in preliminary studies. Similarly, noticeably low drug loading results were obtained with microfluidic-prepared niosomes (data not shown). In addition, no improvement on encapsulation efficiency can be seen in formulations with using higher drug concentrations and remote drug loading techniques (vortex and water bath) used. The low %EE revealed the occurrence of drug leakage as a result of the drug characteristics to remain dissolved in the acidic medium and subsequently be removed during dialysis. Therefore, the highest encapsulation efficiency obtained in microfluidic-prepared niosomes with Cremophor^®^ ELP among other formulations was chosen for drug release study. The manufacture of the formulation was optimised to prepare empty pre-formed niosomes followed by mixing with cinnarizine solution in 0.1 M HCl solution by using water bath shaker.

**Figure 10:**
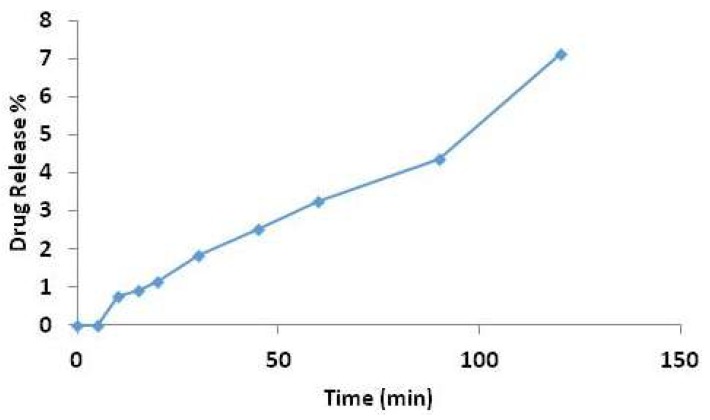
Drug release data for microfluidic prepared niosomes with Cremophor^®^ ELP as a co-surfactant.

The drug release at pre-determined intervals was measured for the optimised formulation in this study. The in vitro drug release of cinnarizine from microfluidic-prepared niosomes (S60: Cho: ELP) showed a noticeably low drug release for up to 2 h (Figure 10). A 50% release of encapsulated cinnarizine was achieved at 24 h (data not shown). The slow release pattern shown suggested there is an interaction between the hydrophobic drug and the amphiphilic niosome vesicles. Niosome has been formulated to improve solubility of various poorly water-soluble drugs aiming to enhance drug dissolution, and hence, a lower dose and less frequency of administration required. Moreover, niosomes show the potential of protecting encapsulated cinnarizine within their vesicles in the acidic environment (0.1 M HCl solution, pH 1.2) and releasing the drug slowly in the target absorption site (stomach) at the same time. Empty pre-formed niosomes prepared by microfluidic method ensures the uniformity of niosomes and the homogeneity (low PDI value) of the formulation to enhance predicted drug release. The prepared niosomes show also promise to incorporate potent drugs for which the small amount of drug released is being efficient and effective to produce the drug effect.

## 4. Conclusions

In this study, the niosome characteristics influenced by preparation methods and the presence of cinnarizine as a model for poorly water-soluble drug have been investigated. Niosome vesicles comprised of equimolar of Span^®^ 60 and cholesterol with different co-surfactants were successfully prepared using both conventional thin-film hydration and microfluidic methods. The characterisation studies demonstrated that the drug and co-surfactants have effects on niosomes characteristics prepared by both thin-film hydration and microfluidic methods. Niosomes have shown potential for prolonged release of cinnarizine in which the current multiple dosing can be reduced. Furthermore, a smaller dose will be required to be taken as niosomes are capable of delivering to targeted absorption site for higher bioavailability. The type of media used during niosome preparation should be taken into consideration particularly for poorly water soluble drugs. Additionally, the microfluidic system produced good quality vesicles even without co-surfactant (F1 and F2) and this method verified also the simplified preparation step for desirable and consistent physiochemical properties of niosomes. Therefore, it is a promising technique not only for liposomes as reported in literature but also for niosomes as reported from this current research. This will open more doors for researchers including authors of this research to apply microfluidic technique for niosomal production. Especially, it can be used for industrial production of plain niosomes which can adapt different drugs as the niosomes properties will mostly not be changed with addition of drugs at the same time ensuring the formulation homogeneity and predicted release profiles.

## Figures and Tables

**Table 1:** Compositions of niosomal formulations (initial total lipid content of 10 mg/mL and initial drug content of 0.5 mg/mL).

Formulation Code	Composition (mg)	% Molar Ratio	Cinnarizine	Total Lipid Content (mg)
F1	Span^®^ 60: cholesterol 105.4:94.6	50:50	−	200
F2	Span^®^ 60: cholesterol 105.4:94.6	50:50	+	200
F3	Span^®^ 60: cholesterol: Cremophor^®^ ELP 73.0:65.6:61.4	45:45:10	−	200
F4	Span^®^ 60: cholesterol: Cremophor^®^ ELP 73.0:65.6:61.4	45:45:10	+	200
F5	Span^®^ 60: cholesterol: Cremophor^®^ RH40 61.6:55.2:83.2	45:45:10	−	200
F6	Span^®^ 60: cholesterol: Cremophor^®^ RH40 61.6:55.2:83.2	45:45:10	+	200
F7	Span^®^ 60: cholesterol: Solutol^®^ HS15 81.8:73.2:45.0	45:45:10	−	200
F8	Span^®^ 60: cholesterol: Solutol^®^ HS15 81.8:73.2:45.0	45:45:10	+	200

**Table 2:** Gradient system of mobile phases.

Time (min)	%A	%B
0	70	30
0.5	70	30
3	10	90
3.5	10	90
3.6	70	30
5	70	30

**Table 3:** Niosomes prepared by thin-film hydration method. * No niosomes formed or large aggregates produced; ** no data obtained. For formulation composition, refer to Table 1.

Formulation	z-Average (nm)	Polydispersity Index (PDI)	Zeta Potential (mV)
S60:Cho −	*	*	*
S60:Cho +	*	*	*
S60:Cho:ELP −	1256.3 ± 59.9	0.759 ± 0.110	**
S60:Cho:ELP +	3701.0 ± 414.6	0.518 ± 0.074	−1.82 ± 6.78
S60:Cho:RH40 −	827.5 ± 147.8	0.759 ± 0.128	−0.063 ± 18.5
S60:Cho:RH40 +	1244.0 ± 314.9	0.714 ± 0.106	0.039 ± 18.1
S60:Cho:HS15 −	5330.3 ± 1348.2	0.262 ± 0.223	−0.199 ± 20.1
S60:Cho:HS15 +	7320.7 ± 675.2	0.933 ± 0.115	0.118 ± 16.5

**Table 4:** Size of niosomes prepared by microfluidic method. For formulation composition, refer to Table 1.

Formulation	z-Average (nm)	PDI	ZP (mV)
S60:Cho −	155.6 ± 0.4	0.059 ± 0.011	−0.474 ± 9.7
S60:Cho +	217.9 ± 0.9	0.053 ± 0.009	−1.266 ± 9.6
S60:Cho:ELP −	350.3 ± 7.1	0.208 ± 0.012	5.272 ± 3.5
S60:Cho:ELP +	355.3 ± 1.5	0.202 ± 0.021	−4.486 ± 5.3
S60:Cho:RH40 −	224.7 ± 2.2	0.103 ± 0.019	−3.921 ± 4.3
S60:Cho:RH40 +	172.2 ± 0.4	0.209 ± 0.007	−1.803 ± 4.8
S60:Cho:HS15 −	310.8 ± 0.6	0.032 ± 0.051	−4.801 ± 3.6
S60:Cho:HS15 +	303.7 ± 1.9	0.011 ± 0.010	−0.764 ± 14.3

**Table 5:** Principal peaks with assignments of cinnarizine pure drug.

Frequency (cm^−1^)	Assignments
2959	CH stretching (aromatic, alkene, mono-substituted)
2936	CH stretch (aliphatic, alkane)
1597	C=C (aromatic stretch)
1490, 1448	CH_2_ (alkane)
1134	C-N stretching
999, 962	=C-H out of plane (out-of-plane) (aromatic, alkene)

**Table 6:** %EE ^a^ Microfluidic niosomes; ^b^ Thin-film hydration niosomes.

Formulation	%EE ^a^	%EE ^b^
S60:Cho:ELP +	8.9	19.5
S60:Cho:RH40 +	0.37	10.8
S60:Cho:HS15 +	0.53	8.3
